# THBS1, a fatty acid-related metabolic gene, can promote the development of laryngeal cancer

**DOI:** 10.1038/s41598-022-23500-6

**Published:** 2022-11-05

**Authors:** Fei-Hong Ji, Xin-Guang Qiu

**Affiliations:** Thyroid Surgery, the First Affiliated Hospital of Zhengzhou University, Zhengzhou, Henan China

**Keywords:** Cancer, Cell biology, Computational biology and bioinformatics, Biomarkers, Oncology

## Abstract

Laryngeal cancer is the second most prevalent head and neck tumor and it is one of the most common malignancies of the upper respiratory tract. Fatty acid metabolism affects cancer cell biology in several ways, and alterations in fatty acid metabolism are characteristic of both tumorigenesis and metastasis. Despite advances in laryngeal cancer diagnosis and treatment over the years, there has been no significant improvement in survival or mortality. Studying the role of fatty acid metabolism-related genes in laryngeal cancer will facilitate our search for valuable biomarkers to guide prognostic management and treatment selection. We constructed a prognostic risk score model for fatty acid metabolism-related genes by downloading and analyzing laryngeal cancers from the TCGA and GEO databases. We predicted survival outcomes of laryngeal cancer patients using a prognostic risk score model of fatty acid metabolism-related genes and analyzed the resistance of laryngeal cancer in different individuals to multiple drugs. In addition, the relationship between the prognostic risk score model and cellular infiltration characteristics of the tumor microenvironment were investigated. Through the prognostic risk scoring model, the genes with risk-prompting effect and related to prognosis were screened out for further research. Through the study of gene expression levels in the TCGA database, we screened out 120 differentially expressed fatty acid metabolism genes. LASSO-Cox and Cox regression analyses identified nine genes associated with prognosis to construct a prognostic risk score model for genes related to fatty acid metabolism. Both TCGA and GEO confirmed that samples in the high-risk score group had a worse prognosis than those in the low-risk score group. We found significant differences between the high-risk and low-risk groups for 22 drugs (*P* < 0.05). In addition, we found differences in immune cell infiltration between the different risk score groups. Finally, through the risk assessment model, combined with multiple databases, THBS1, a high-risk and prognosis-related gene, was screened. We also found that THBS1 could promote the migration, invasion and proliferation of laryngeal cancer cells by constructing THBS1 knockout cell lines. In our study, we identified key fatty acid-related genes differentially expressed in laryngeal carcinoma that can be used to adequately predict prognosis using a comprehensive bioinformatic experimental approach. It was also found that THBS1, a high-risk and prognosis-related gene, may regulate the occurrence and development of laryngeal cancer through fatty acid metabolism, which has further helped us to explore the role of fatty acid metabolism genes in laryngeal cancer.

## Introduction

Head and neck tumors are among the most common malignancies, with laryngeal cancer being the second most prevalent head and neck cancer and one of the most common malignancies of the upper respiratory tract^1^. The 5-year survival rate for patients with laryngeal cancer is approximately 60%^2,3^. Despite advancements in the diagnosis and treatment of laryngeal cancer over the years, survival and mortality have not significantly improved^4^. Cancer cell invasion, recurrence, and metastasis are crucial causes of surgical failure and death^5^. At present, there are no effective targeted drugs for laryngeal cancer^6^. Therefore, identifying ideal molecular markers is important to improve the clinical diagnosis and treatment of laryngeal cancer.

Fatty acid metabolism affects cancer cell biology, especially the synthesis of lipid building blocks of membranes (glycerophospholipids) and signaling intermediates^7^. Fatty acid metabolism includes anabolic and catabolic processes required for energy homeostasis, along with the formation of metabolic intermediates necessary to maintain cell membrane structure and function, cell signaling, and stored energy^8,9^. Cancer cells can acquire fatty acids from various extracellular and intracellular sources, and alterations in fatty acid metabolism are characteristics of tumorigenesis and metastasis^10^. Altered fatty acid metabolism is one of the crucial potential mechanisms underlying the altered behavior of numerous cancer types in patients with obesity, type 2 diabetes, and metabolic syndrome^11^.

In recent years, fatty acid metabolism has been increasingly recognized to profoundly affect tumor progression through β-oxidation and the substantial availability of glycerophospholipid synthesis and not only ATP production^12^. Specifically, this includes maintaining homeostasis of fatty acids under redox stress, thereby preventing ferroptosis, and affecting membrane fluidity and permeability to facilitate motility and metastasis^13^. However, to the best of our knowledge, genes related to fatty acid metabolism in laryngeal cancer have not been systematically investigated. Therefore, the present study aimed to analyze genomic information from The Cancer Genome Atlas (TCGA) database and Gene Expression Omnibus (GEO) laryngeal cancer samples, comprehensively assess the metabolic pattern of fatty acids, and construct a fatty acid prognostic risk-scoring model. We predicted the survival outcome of patients with laryngeal cancer using a prognostic risk score model for genes related to fatty acid metabolism and analyzed their resistance to multiple drugs. In addition, we investigated the relationship between the prognostic risk-scoring model and characteristics of tumor microenvironment cell infiltration.

## Materials and methods

### Data screening and processing

The datasets generated during the current study are available in those publicly available datasets. The 264 fatty acid metabolism-related genes (FAMRGs) were obtained from the GeneCards database (HADb, https://www.genecards.org/ ). Transcriptomic data and clinical characteristics of laryngeal cancer samples were obtained from the TCGA (https://portal.gdc.cancer.gov/ ). A separate laryngeal cancer dataset GSE25727 was obtained from GEO (https://www.ncbi.nlm.nih.gov/geo/query/acc.cgi?acc=GSE25727) and was selected as the validation cohort. The patients involved in the database have received ethical approval for this publicly available database. Users can download relevant data for free research and publish relevant articles. Our study is based on public source data, so there are no ethical issues and other conflicts of interest.

The Limma package in R statistical software was applied to evaluate differentially expressed FAMRGs between laryngeal cancer and non-tumor samples. genes exhibiting at |logFC|> 0.585, false discovery rate (FDR) < 0.05, *P* < 0.05 were selected as differentially expressed FAMRGs. TCGA and GEO belong to public databases.

For the human studies covered in this study, data and information were obtained from the above publicly available databases (ethical approval is attributed to the above publicly available databases). The cellular and other experiments involved in the follow-up study were approved by the Ethics Committee of the First Affiliated Hospital of Zhengzhou University (the ethical review permit number for this study is 2021-KY-0202-002).

### Analysis of differential expression of FAMRGs

Differential expression of FAMRGs were obtained by comparing 12 non-tumor and 111 laryngeal cancer tissues in TCGA and the filter criteria was |logFC|> 0.585, *P* < 0.05and FDR < 0.05. Differential expression of FAMRGs were then utilized to perform Kyoto Encyclopedia of Genes and Genomes (KEGG) and Gene Ontology (GO) analysis. Protein–protein interaction (PPI) was conducted by the STRING database and Cytoscape. Gene features were constructed as follows: overlapping FAMRGs were identified by an unsupervised clustering algorithm, and then the patients were classified into different groups. We used a consistent clustering algorithm to ensure the number of clusters and their stability. We then determined the prognostic value of each gene by building a univariate Cox regression model, and based on this, selected genes with significant prognostic value for further analysis. Finally, the gene profile related to fatty acid metabolism was constructed by principal component analysis (PCA).

### Construction and validation of the prognostic signature

We identified prognostic features by using multivariate Cox regression analysis to find FAMRGs for laryngeal cancer. Kaplan–Meier (K-M) and Receiver Operation Characteristic (ROC) curves were used to determine the prognostic value of the feature. the GEO dataset GSE25727 was used to validate the prognostic features. Univariate and multivariate Cox analyses were used to identify risk factors. Correlation analysis between risk scores and clinical characteristics and construction of nomograms were performed based on clinicopathological parameters. Calibration plots were used to compare the agreement between predicted and actual probabilities of 1/3/5-year survival.

### Gene set variation analysis (GSVA) and functional annotation

To study the biological processes and pathways under distinct fatty acid metabolism-related patterns, the "c2.cp.kegg.v7.4.symbols.gmt" gene set of GSVA was obtained. p-values less than 0.05 indicate significant enrichment of biological processes and pathways (http://www.gsea-msigdb.org/gsea/index.jsp). Functional annotation of FAMRGs was analyzed by the "clusterProfiler" package. The adjusted p-values less than 0.05 were considered statistically significant.

### Immune cell infiltration analysis

The proportion of 22 immune cell types was derived for each laryngeal cancer sample from TCGA by the R package "CIBERSORT" (https://cibersort.stanford.edu/). Only samples with *P* < 0.05 were considered for further analysis to compare the differences in the levels of tumor-infiltrating immune cells (TICs) between the low and high prognosis groups.

### Cell cultures

The human laryngeal cancer cell lines like LCC and TU686 with STR identification were cultured in 1640 (Gibco, Detroit, MI, USA) with 10% fetal bovine serum (Gibco, Detroit, MI, USA), penicillin (100 units/ml, (Grand Island, NY)) and streptomycin (100 μg/ml, (Grand Island, NY)) in 95% air and 5% CO_2_ at 37 °C. STR fingerprint authentications of LCC and TU686 cells are available upon request.

### Quantitative real-time PCR (qRT-PCR)

A total RNA extraction kit (Vazyme, Nanjing, China) was used to collect total RNA from LCC and TU686 cells. The PrimeScript RT reagent kit (Vazyme, Nanjing, China) was employed for reverse transcription of RNA into cDNA. GAPDH served as an internal control. Furthermore, all reactions were carried out based on the following cycle parameters. Specific amplification through dissociation curve analysis was conducted. All data represent the average of triplicates under the calculation of 2^−ΔΔCt^ method.

### Cell proliferation assay

Cell proliferation was valued by the Cell Counting Kit-8 (CCK-8) Assay Kit (Vazyme, Nanjing, China). The KTC-1 and TPC-1 cells were cultured in 96-well plates with 1.5 × 10^3^ cells/well\. A microplate reader was measured in the absorbance at 450 nm. The proportion of relative cell proliferation through the average optical density of each group was calculated. The formula is as follows: (OD treatment/OD control) × 100%.

### Transwell assays

LCC and TU686 cells were inoculated into 24 -well transwell chambers with polycarbonate membrane (353,097) (Corning Life Science, Acton, MA) to evaluate invasion capacities under the function of Matrigel (Corning, U.S.) or without it. The upper chamber was injected with cells in a serum-free medium at a density of 4 × 10^5^ cells/well and the outer chamber got filled with the complete-culture medium. After a 12-h culture process, cells adhered to the underside of the chamber for 30 min were fixed by paraformaldehyde, and later got calculated under an optical microscope (Zeiss, Germany).

### Lentivirus vector infection

Lenti-viruses expressing THBS1, THBS1-shRNA (target sequences: gc GCGTGTTTGACATCTTTGA), scrambled shRNA, and vector purchased from Genechem (Shanghai, China). In brief, 3 × 10^4^ cells were seeded into a 6-well plate and then transfected with the specified lenti-virus using HitransG (Genechem, Shanghai, China). In addition, infected cells were selected by 2.5 μg/ml puromycin (MCE, USA) for > 14 days, and verified by RT-qPCR.

### Statistical analysis

Statistical analyses were conducted with software R (v4.1.1) , GraphPad Prism 8.0 (GraphPad, Inc., USA) and SPSS 22.0 (SPSS, Inc., USA) software. All statistical tests followed the two-way path The difference between groups were tested by one-way analysis of variance (ANOVA). Representative data was demonstrated as mean ± SD. In addition, correlation analysis of gene expression was carried out with linear regression. *P* < 0.05 was treated with statistical significance.

### Ethics approval and consent to participate

The datasets generated during the current study are available in those publicly available datasets. The 264 fatty acid metabolism-related genes (FAMRGs) were obtained from the GeneCards database (HADb, https://www.genecards.org/ ). Transcriptomic data and clinical characteristics of laryngeal cancer samples were obtained from the TCGA (https://portal.gdc.cancer.gov/ ). A separate laryngeal cancer dataset GSE25727 was obtained from GEO (https://www.ncbi.nlm.nih.gov/geo/query/acc.cgi?acc=GSE25727) and was selected as the validation cohort. The patients involved in the database have received ethical approval for this publicly available database. Users can download relevant data for free research and publish relevant articles. Our study is based on public source data, so there are no ethical issues and other conflicts of interest.

### Human and animal rights

For the human studies covered in this study, data and information were obtained from the above publicly available databases (ethical approval is attributed to the above publicly available databases). The cellular and other experiments involved in the follow-up study were approved by the Ethics Committee of the First Affiliated Hospital of Zhengzhou University (the ethical review permit number for this study is 2021-KY-0202–002).

### Consent to publish

All authors agree to the publication of this manuscript.

## Results

### Identification of differential FAMRGs and development of prognostic risk-scoring models in the training set

We obtained transcriptomic data and clinical characteristics of laryngeal cancer samples from the TCGA (https://portal.gdc.cancer.gov/) database, including 111 laryngeal cancer patient samples, 12 non-tumor tissue samples (Supplementary file 1, 2), we downloaded a separate laryngeal cancer dataset from the GEO database, numbered GSE25727 (https://www.ncbi.nlm.nih.gov/geo/query/acc.cgi?acc=GSE25727), which includes 56 laryngeal cancer tumor patient samples (Supplementary file 3).

**Figure 1:**
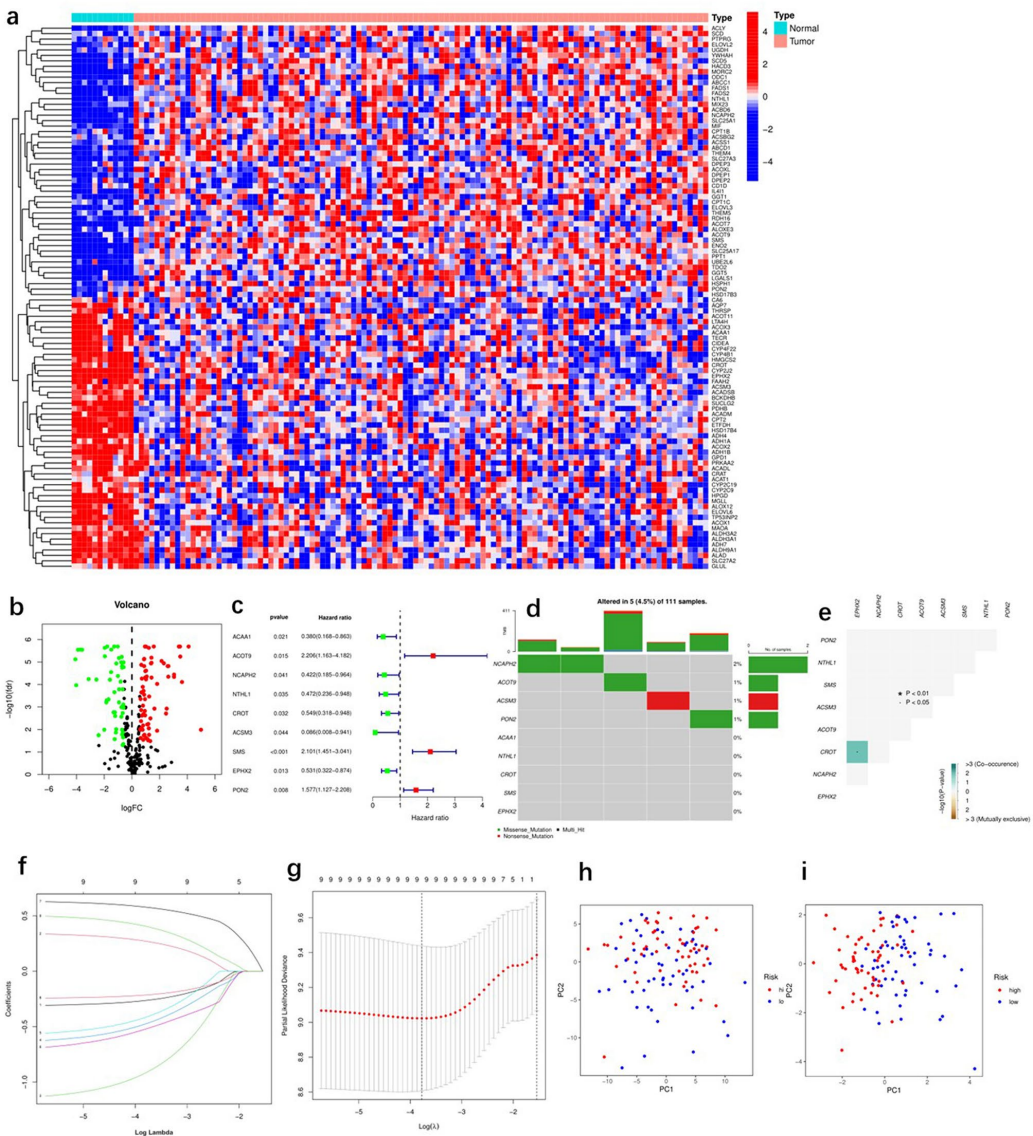
Identification of differential fatty acid metabolism-related genes and developing prognostic risk scoring models in the training set. (**a**) The heat map of 120 differentially expressed fatty acid metabolism-related genes. (**b**) The volcano of 120 differentially expressed fatty acid metabolism-related genes. (**c**) Forrest plot of 9 fatty acid metabolism-related genes related with prognosis. (**d**) The mutation frequency of 9 fatty acid metabolism-related genes in 117 patients with LARYNGEAL CANCER from TCGA cohort. (**e**) The mutation co-occurrence and exclusion analyses for 9 fatty acid metabolism-related genes. Co-occurrence, green; exclusion, purple. (**f**) LASSO coefficients of the 9 fatty acid metabolism-related genes. (**g**) Identification of genes for development of prognostic risk score model. (**h**) Principal component analysis based on all fatty acid metabolism-related genes in laryngeal cancer. (**i**) Principal component analysis based on fatty acid metabolism risk score to distinguish tumors from normal samples in TCGA cohort. The group marked with blue represented high-risk patients, and the group marked with red represented low-risk patients.

The Gene Cards database defines 264 FAMRGs, among which 120 FAMRGs were selected by comparing the expression of FAMRGs between cancer and non-tumor tissue samples in TCGA (|logFC|> 0.585, FDR < 0.05, *P* < 0.05). A total of 51 and 69 genes were downregulated and upregulated, respectively, in the cancer tissue samples (Fig. 1a,b). The specific information of these 120 fatty acid metabolism genes can be found in the Supplementary file 4. Univariate Cox regression analysis was used for the 120 differentially expressed FAMRGs. Nine genes associated with prognosis were identified (*P* < 0.05) (Fig. 1c). We summarized the somatic mutation profiles of the nine FAMRGs associated with prognosis. Among laryngeal cancer samples in the TCGA database, 5 showed mutations in FAMRGs, with a frequency of 4.5% (Fig. 1d). Further analysis showed mutational co-occurrence between CROT and EPHX2 (Fig. 1e). LASSO-Cox regression analysis was used to determine the number of genes. Finally, nine genes (ACAA1, ACOT9, NCAPH2, NTHL1, CROT, ACSM3, SMS, EPHX2, and PON2) were used to construct a risk-scoring prognostic model (Fig. 1f,g, Table 1), which distinguished laryngeal cancer samples as high or low risk (Fig. 1h,i). The risk score formula used for the sample was as follows: risk score = ACAA1 × (–0.261903442393461) + ACOT9 × (0.269678824841202) + NCAPH2 × (–0.9101717202971) + NTHL1 × (–0.470406427009571) + CROT × (–0.411094477337431) + ACSM3 × (–0.517552679058424) + SMS × (0.577140908056887) + EPHX2 × (–0.21327197630715) + PON2 × (0.403413168237389).

**Table 1:** 9 fatty acid metabolism genes related to prognosis.

id	HR	HR.95L	HR.95H	*P* value
ACAA1	0.380227	0.167609	0.86256	0.020682
ACOT9	2.205899	1.163473	4.182299	0.015357
NCAPH2	0.422246	0.184913	0.964189	0.040705
NTHL1	0.472498	0.235518	0.947931	0.034816
CROT	0.548759	0.317593	0.948183	0.031504
ACSM3	0.085572	0.007782	0.940922	0.044458
SMS	2.100902	1.451273	3.041324	8.38E-05
EPHX2	0.530714	0.322093	0.874459	0.012902
PON2	1.577379	1.126753	2.208226	0.007926

### Predictive value of the fatty acid metabolic-scoring model for survival of patients with laryngeal cancer

**Figure 2:**
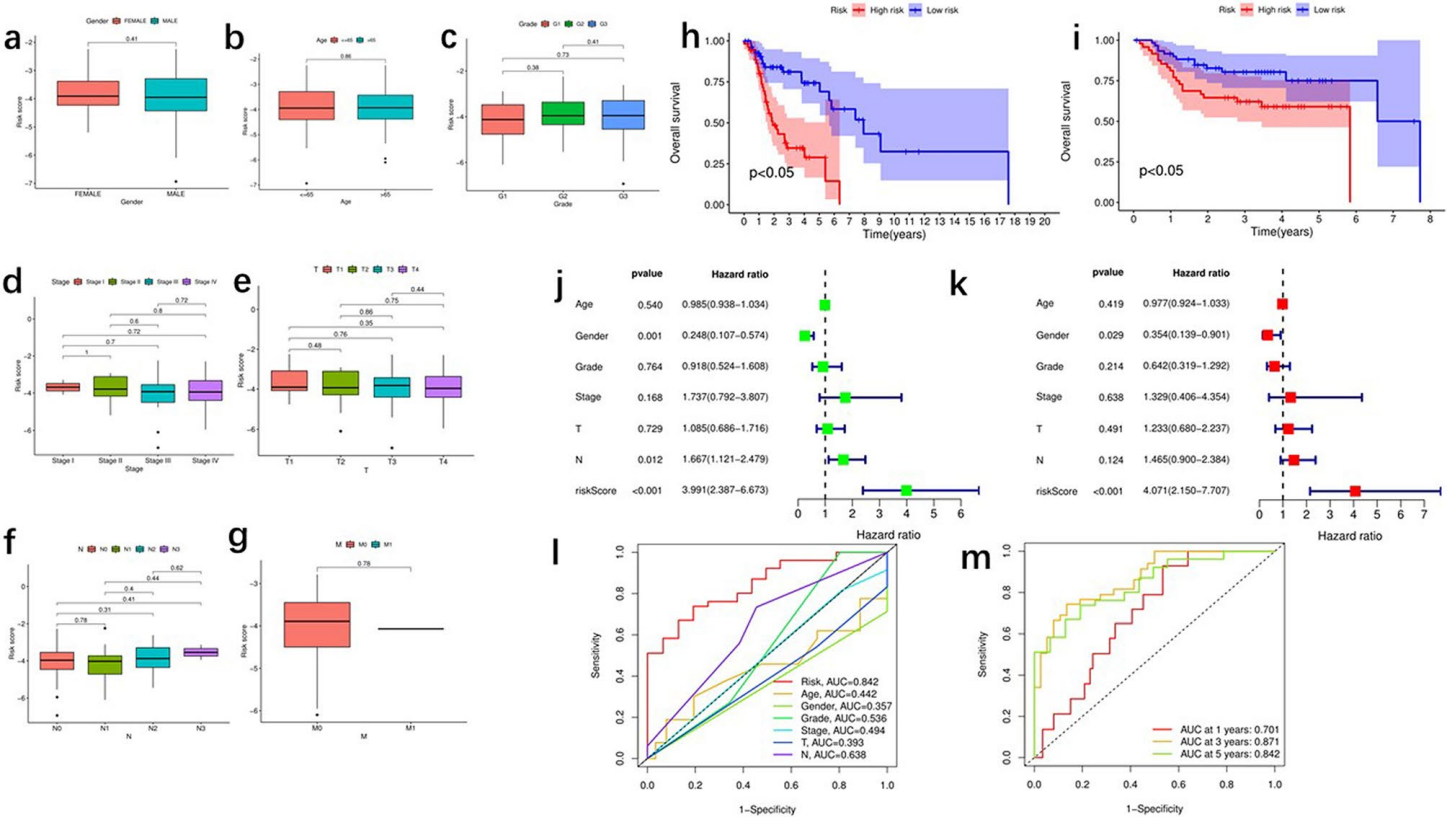
The predictive value of fatty acid metabolism score model in survival status of laryngeal cancer patients. (**a**–**g**) The relationship of risk score and clinicopathological features, including gender (**a**), age (**b**), grade (**c**), stage (**d**), and TNM (**e**–**g**). (**h**–**i**) The comparison of overall survival (OS) between low- and high-risk score groups in the training set and the test set in TCGA datasets and GEO datasets. (**j**–**k**) The forest plot of the univariate and multivariate Cox regression analysis in TCGA cohort. (**l**–**m**) The predictive value of the risk score measured by ROC curves in the training set and the test set.

The critical value was the median value of the risk score in the training set. Based on the above threshold values, the samples were ranked and divided into high-risk score groups (n = 55) and low-risk score groups (n = 56). Age, sex, stage, grade, and tumor, node, metastasis (TNM) malignancy classification risk score distribution were analyzed for the corresponding samples. We found no relationship between the low- and high-risk groups for age, sex, stage, grade, and TNM staging of the sample (Fig. 2a–g). The prognosis was worse in the low-risk score group than in the high-risk score group (*P* < 0.05) (Fig. 2h). GSE25727, the test group samples from the GEO, was divided into low-risk score groups (n = 54) and high-risk score groups (n = 55). Samples in the low-risk score group had a better prognosis than those in the high-risk group (Fig. 2i). Combining the TCGA and GEO databases showed that the prognostic risk score models of the low- and high-risk score groups predicted the overall survival (OS) of laryngeal cancer. In univariate analysis, factors associated with OS included sex (*P* = 0.001), N stage (*P* = 0.012), and risk score (*P* < 0.001); the multivariate analysis showed that factors associated with OS included sex (*P* = 0.029) and risk score (*P* < 0.001) (Fig. 2j,k). The area under the receiver operating characteristic (ROC) curve (AUC) showed a greater predictive value for risk (AUC = 0.842) than for single indicators, such as age (AUC = 0.442), sex (AUC = 0.357), class (AUC = 0.536), and T (AUC = 0.393) and N (AUC = 0.638) stages (Fig. 2l). Furthermore, we plotted a time-related subject ROC curve at 1, 3, and 5 years to verify the prognostic risk score model accuracy (Fig. 2m).

### Predictive value of OS in TCGA cohort patients by combining fatty acid metabolism scores with clinicopathological features

**Figure 3:**
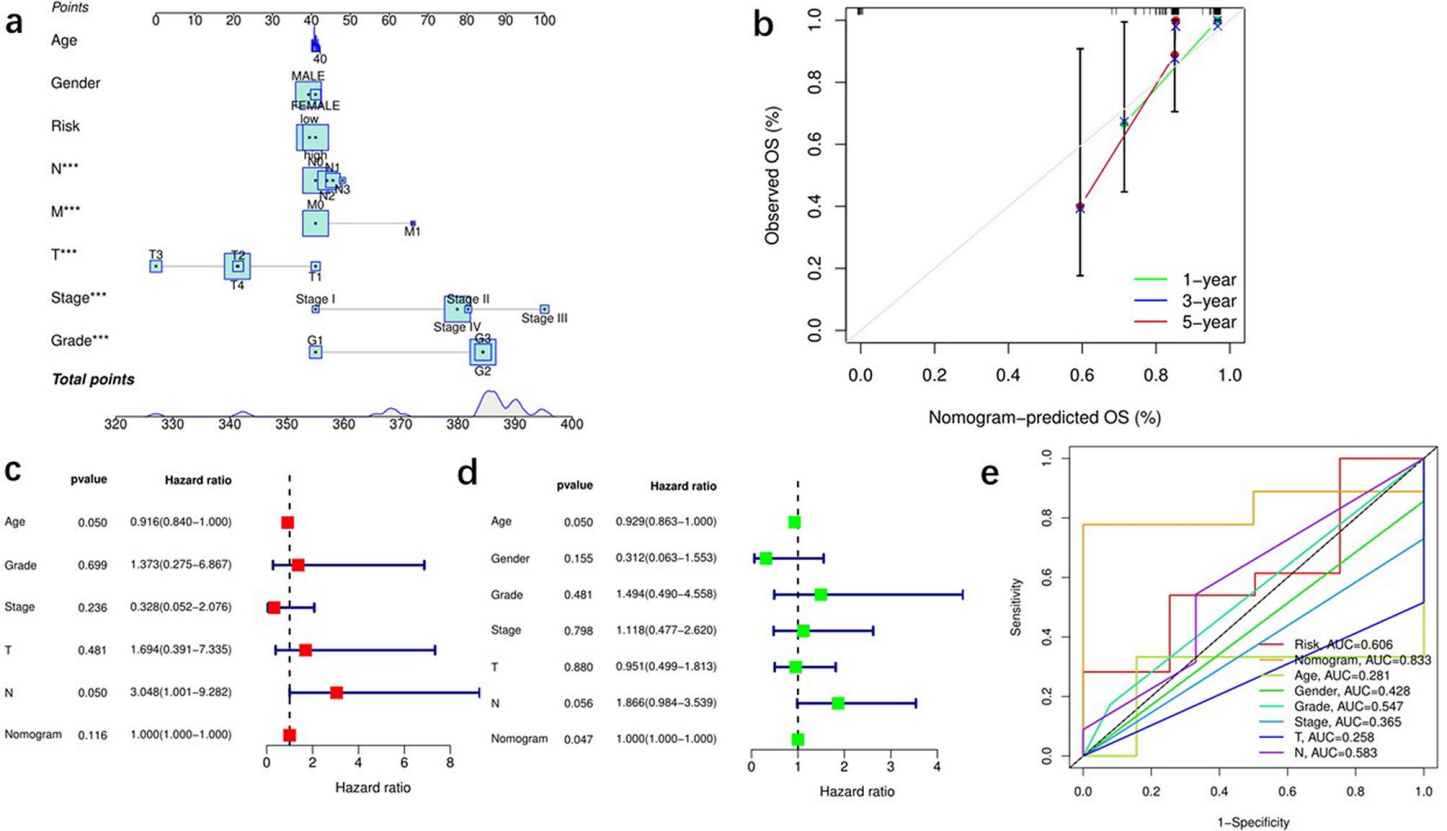
The predictive value of fatty acid metabolism score in combination with clinical pathological characteristics in OS of patients from TCGA cohort. (**a**) Nomogram predicting OS of patients from TCGA cohort. (**b**) The calibration plots of the nomogram. The x axis is nomogram-predicted survival, and the y axis is actual survival. (**c**) Multivariate Cox regression analysis of the nomogram. (**d**) Univariate Cox regression analysis of the nomogram. (**e**) ROC curves for fatty acid metabolism score and clinical pathological characteristics.

To predict OS for laryngeal cancer samples, we constructed a prognostic risk score model, a column line plot combining age, sex, pathological stage, and T and N stages (Fig. 3a). The 1-, 3-, and 5-year calibration curves illustrate that column line graphs can accurately predict OS in patients with laryngeal cancer (Fig. 3b). The results of the univariate Cox regression (Fig. 3c) and multivariate Cox regression (Fig. 3d) analyses showed that in the prognostic risk score model, age was an independent prognostic indicator. The AUC showed that the column line graph (AUC = 0.833) had a greater prognostic value than age (AUC = 0.281), sex (AUC = 0.428), and grade (AUC = 0.547) (Fig. 3e).

### The roles of fatty acid metabolism models in chemotherapy

**Figure 4:**
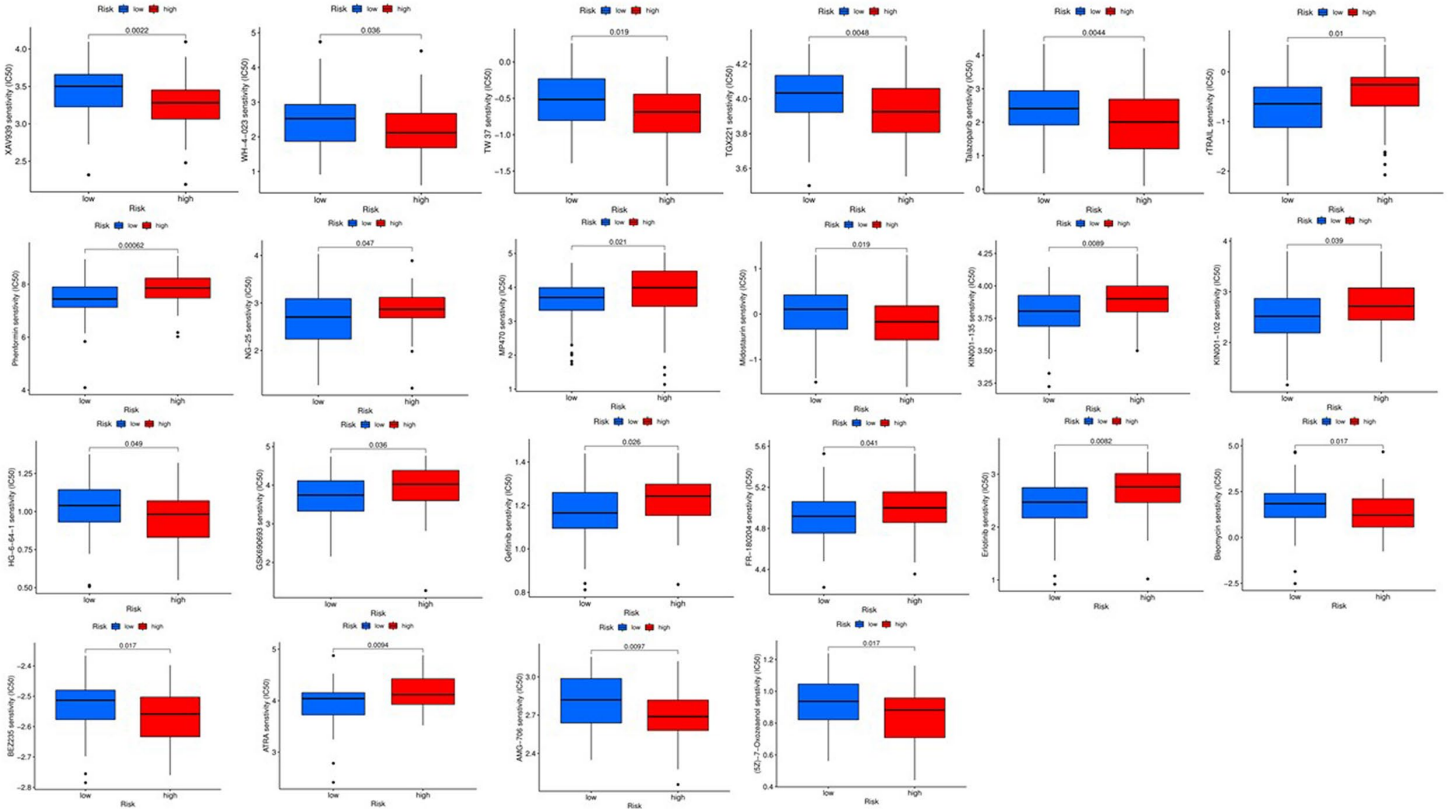
The differences of response to 22 drugs between low- and high-risk score groups.

**Figure 5:**
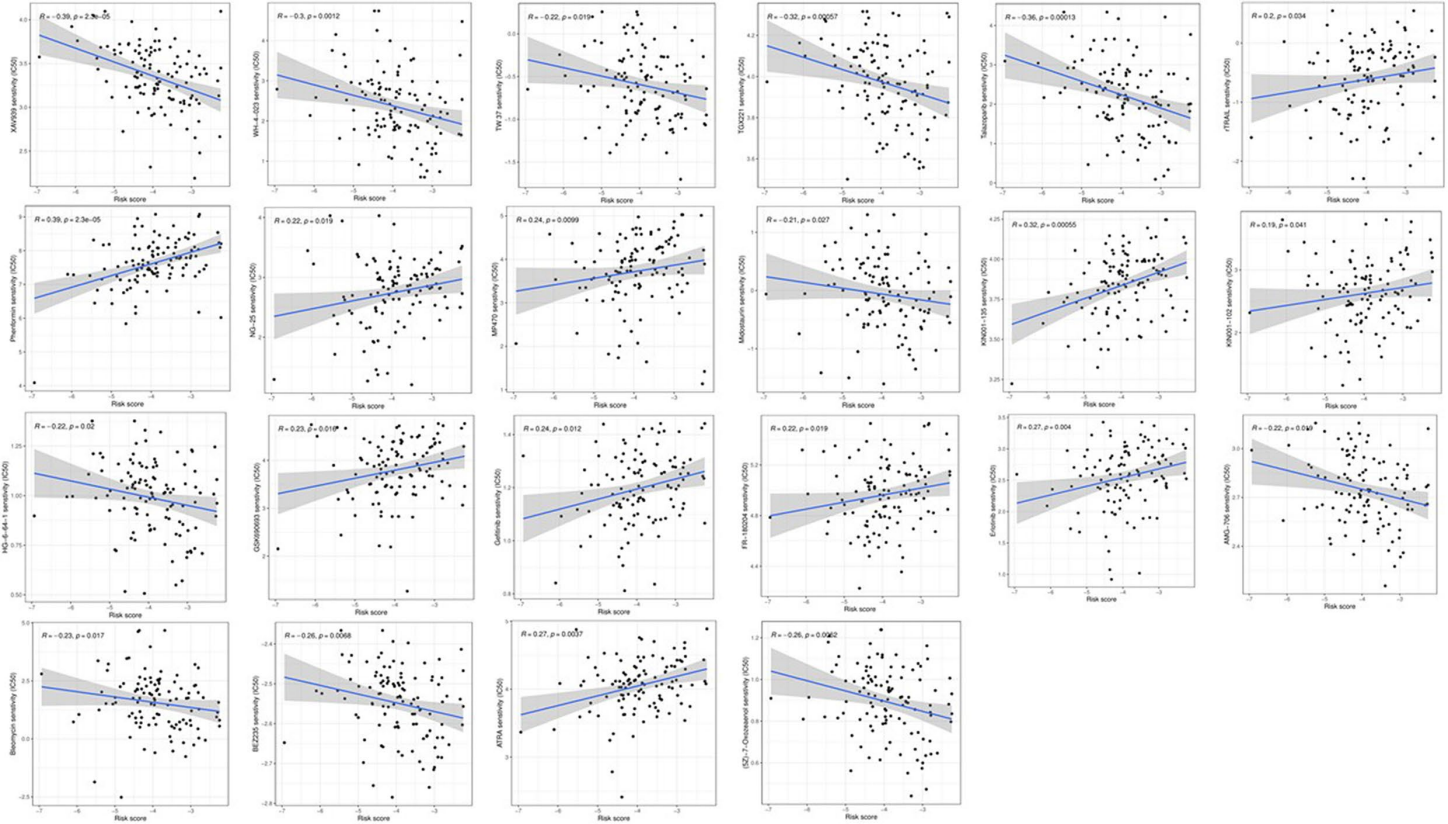
The correlation between risk scores of patients and estimated IC50 value of 22 drugs.

**Figure 6:**
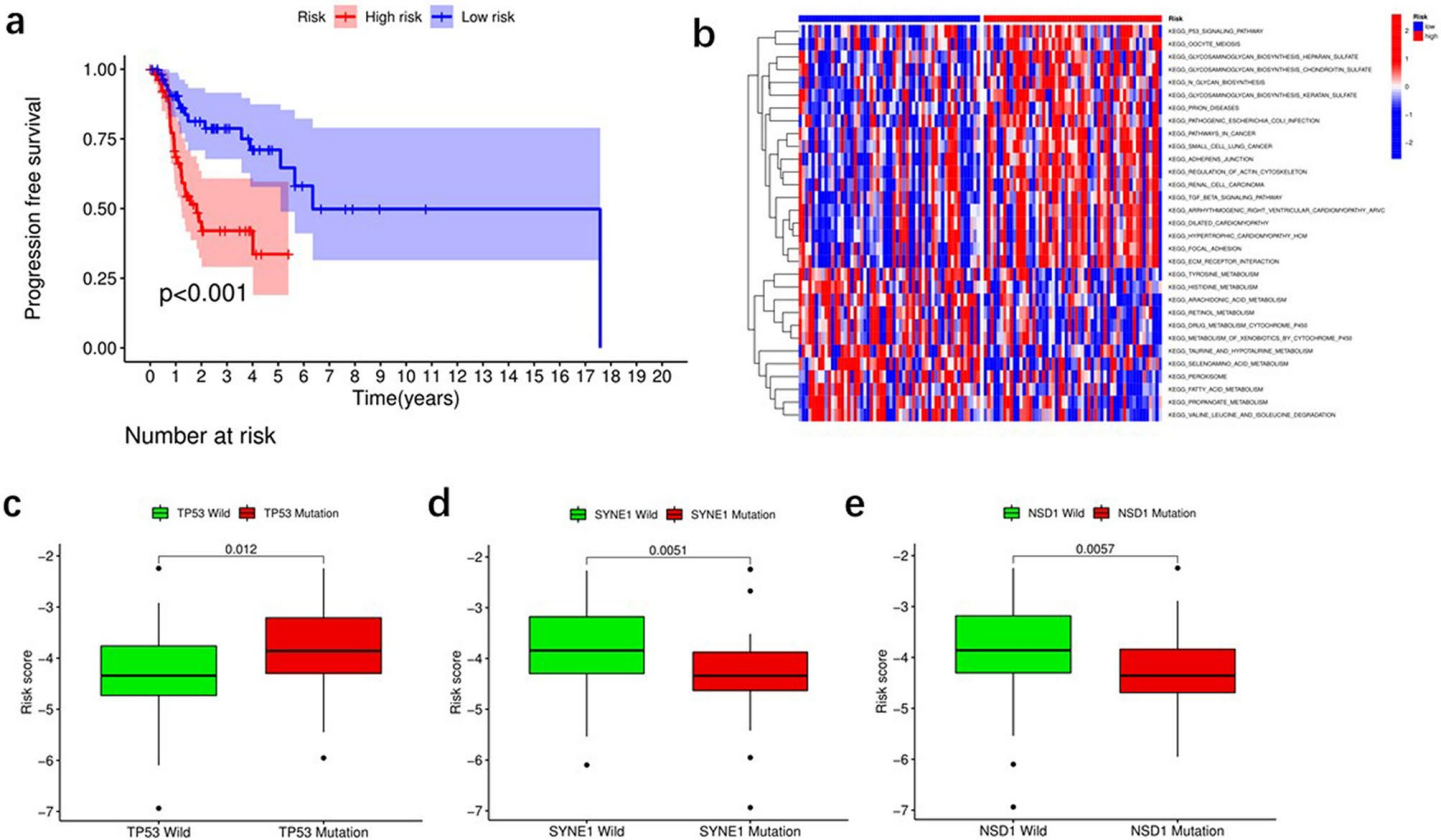
Fatty acid metabolism model in the role of chemotherapy. (**a**) The comparison of disease-free survival (DFS) between low- and high-risk score groups in the GEO cohort. (**b**) The heatmap of GSVA enrichment between low- and high-risk score groups. (**c**–**e**) Differences in fatty metabolism score among different of molecule subtypes, including TP53 mutation (**c**), SYNE1 mutation (**d**), and NSD1 status (**e**).

As risk score is usually related to poor prognosis, we explored the relationship between risk score and chemoresistance. We predicted the treatment response of multiple drugs in TCGA laryngeal cancer data by calculating the half-maximal inhibitory concentration (IC50). We identified 22 drugs that differed between the low-risk and high-risk laryngeal cancer groups (Figs. 4 and 5). It is well known that there is a strong association between chemotherapy sensitivity and risk scores. In GSE25727, a GEO dataset, there is a great correlation between chemotherapy sensitivity and risk score based on recurrence-free survival (RFS) (Fig. 6a). We used GSVA enrichment from the Molecular Signature Database to explore the biological behavior of both groups. Notably, most metabolic pathways were enriched in the low-risk score group, including fatty acid metabolism (Fig. 6b). In addition, patients with tumor protein p53 (TP53) had a higher risk score (*P* = 0.012) (Fig. 6c), and patients with spectrin repeat-containing nuclear envelope protein 1 (SYNE1) (*P* = 0.0051) (Fig. 6d) and nuclear receptor-binding SET domain protein 1 (NSD1) (*P* = 0.0057) (Fig. 6e) containing spectral repeats had a lower risk score.

### The roles of fatty acid metabolism models in immunotherapy

**Figure 7:**
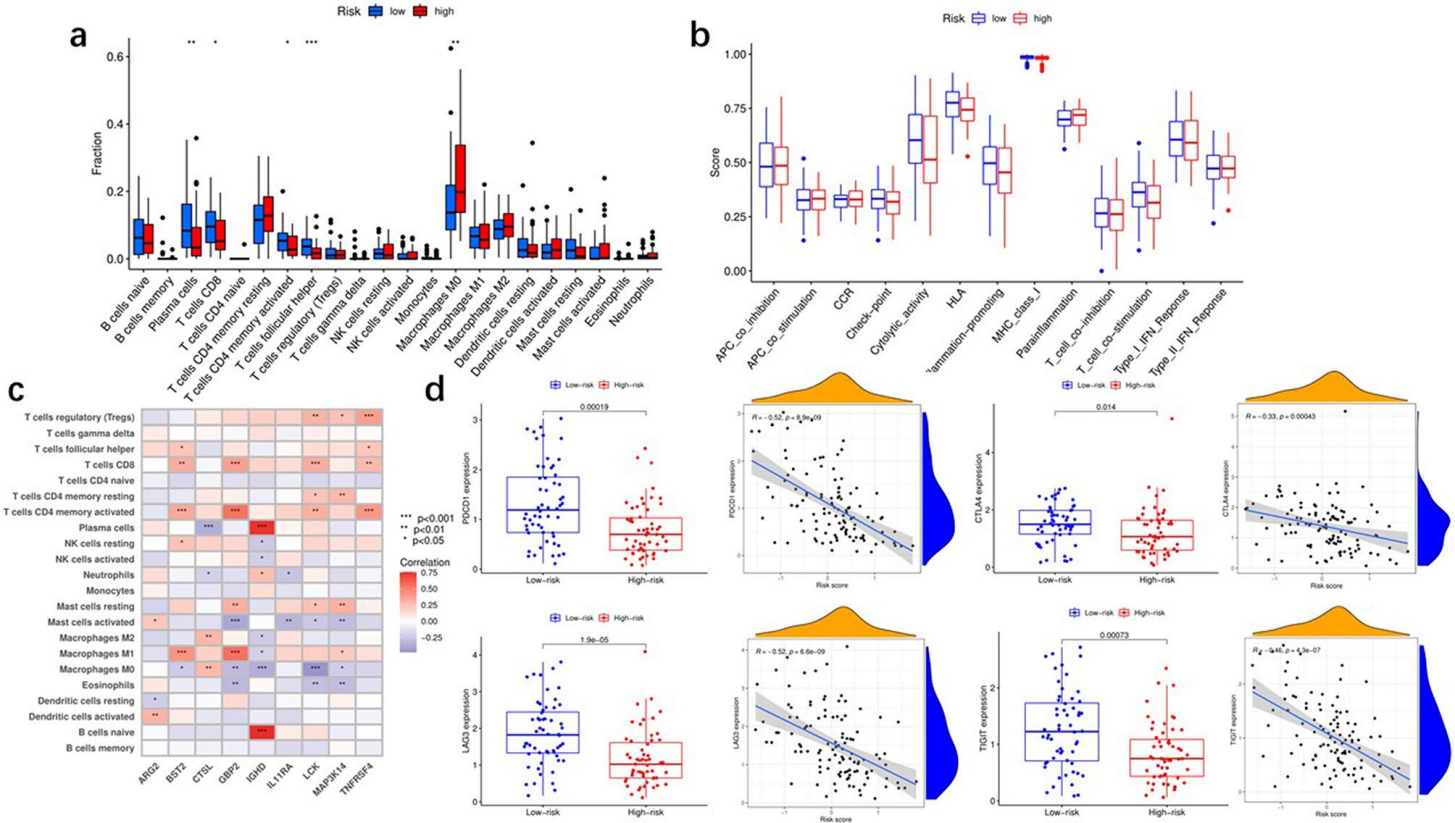
Fatty acid metabolism model in the role of immunotherapy. (**a**) The immunity infiltration difference between high-risk score and low-risk score. (**b**) The known function associated with immunity regulation difference between patients with high-risk score and low-risk score. (**c**) Correlation between TME infiltration cells and fatty acid metabolism genes associated with prognosis. (**d**) The immunity- checkpoint related gene expression difference in high score group and low score group.

We evaluated the variation in immune cell infiltration between the high- and low-risk score groups in the TCGA. High-risk score groups have very high infiltration of immunosuppressive cells (T follicular helper cells, plasma cells, memory CD4-activated T cells, and CD8 + T cells), while macrophages M0 were enriched in the low-risk group (Fig. 7a). Furthermore, we analyzed whether there was a difference between patients in the low-risk group for immunosuppression and those in the high-risk group for developing a response to immunotherapy (Fig. 7b). We also analyzed the correlation between immunosuppressive cell infiltration and the nine prognosis-related genes associated with fatty acid metabolism (Fig. 7c). In addition, we assessed the differences in immune checkpoint-related gene expression between the high-risk and low-risk subgroups in the TCGA cohort in response to treatment with PDCD1, LAG3, CTLA4, and TIGHT. We found that laryngeal cancer samples in the low-risk score group were more sensitive to PDCD1, LAG3, CTLA4, and TIGHT (Fig. 7d).

### PPI network of FAMEGs in the high-risk and low-risk score groups

**Figure 8:**
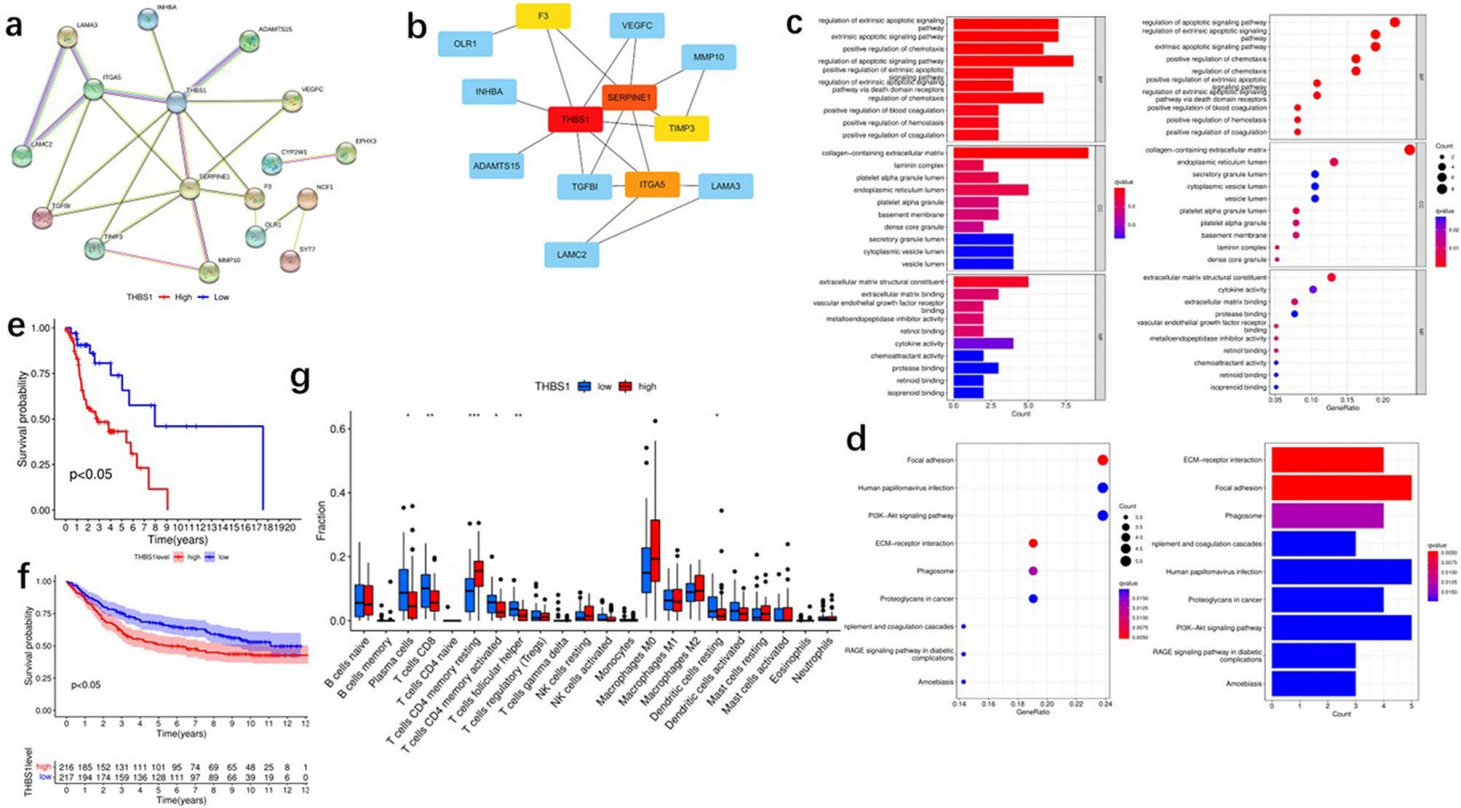
Protein–protein interaction (PPI) network of FAMEGs in the low- risk and high-risk score groups (**a**) PPI network processed by Cytoscape (red): FAMEGs that expressed highly in the high-risk score group; blue: FAMEGs that expressed highly in the low-risk score group. (**b**) Top 5 hub genes selected by cytoHubba. (**c**–**d**) The results of GO and KEGG enrichment analysis on top 5 hub genes. **e.** Survival analysis for subgroup patients stratified for Top 5 hub genes. (**f**) The abundance of each TME-infiltrating cell in patients with high and low THBS1 mRNA expression.

In the previous content, we validated the value of the risk model composed of nine fatty acid metabolism genes from multiple perspectives. We used this risk score formula to screen 24 high-risk genes in the TCGA and GEO databases (Table 2). We processed these 24 genes through Cytoscape software to display the PPI network data, using the STRING online database (https://www.string-db.org/) to analyze the expression profiles of FAMEGs in high-risk and low-risk groups in TCGA score groups (Fig. 8a). We used the cytoHubba plugin to identify the following top five hub genes from FAMEGs: F3, Thrombospondin-1 (THBS1), SERPINE1, TIMP3, and ITGA5 (Fig. 8b). Moreover, we performed KEGG and GO analysis on five different hub genes to better understand their functions (Fig. 8c,d). We performed prognostic analysis on these 5 high-risk genes through TCGA and GEO databases and found that THBS1 was associated with poor prognosis in both TCGA (Fig. 8e) and GEO (Fig. 8f) databases(*P* < 0.05). We used the median THBS1 expression value as the cutoff value and explored the specific differences in tumor microenvironment immune cell infiltration between patients with low and high THBS1 expression. Furthermore, patients with tumors having high THBS1 expression showed significantly increased infiltration in memory CD4-activated T-cell resting compared with patients with tumors having low expression; meanwhile, patients with tumors having low THBS1 expression showed markedly increased infiltration of plasma cells, follicular helper T cells, memory CD4-activated T cells, CD8 + T cells, and dendritic cells compared with patients with tumors having high expression (Fig. 8g).

**Table 2:** 24 high-risk genes were screened out by the risk assessment formula.

gene	lowMean	highMean	logFC	*P* value	fdr
MMP10	41.29399	135.4936	1.714221	0.001191	0.047846
MT1L	3.732991	11.46682	1.619061	0.000355	0.03129
LINC00460	0.625789	1.729305	1.466444	0.000496	0.033594
SCG2	0.8461	2.322977	1.457075	0.00029	0.027597
TGFBI	54.49717	144.2908	1.404726	0.000507	0.033915
INHBA	8.49577	21.98665	1.371811	2.44E-05	0.012852
ADAMTS15	1.934269	4.842065	1.323834	0.00027	0.027083
VEGFC	4.337076	10.78428	1.314135	0.000283	0.027214
SYT7	2.770026	6.408786	1.210152	0.000317	0.029193
SERPINE1	74.24075	169.9663	1.194966	0.0001	0.02038
THBS1	22.65244	51.83526	1.194267	1.19E-05	0.008779
F3	33.30834	74.47115	1.160798	0.000645	0.037931
LAMA3	22.46598	50.1633	1.15889	0.000464	0.033594
SRPX	5.130644	11.32366	1.142129	0.001191	0.047846
EVA1A	1.770387	3.892366	1.136583	2.51E-05	0.012852
TIMP3	1.216318	2.629544	1.112293	0.000566	0.036703
LAMC2	155.1661	329.9548	1.088455	0.000416	0.032895
SPOCK1	3.373029	7.152242	1.084351	0.000475	0.033594
OLR1	1.73428	3.625027	1.063655	0.000967	0.045455
CLMP	3.049389	6.372341	1.063303	0.000852	0.043258
ITGA5	18.619	38.22299	1.037665	4.65E-06	0.007156
HAS2	1.773876	3.61535	1.02723	0.000332	0.029782
TENM3	1.282331	2.595752	1.017384	0.000363	0.03129
BCAT1	3.0442	6.121697	1.007868	0.00075	0.040758

### THBS1 promotes laryngeal cancer tumor cell migration, invasion, and proliferation

**Figure 9:**
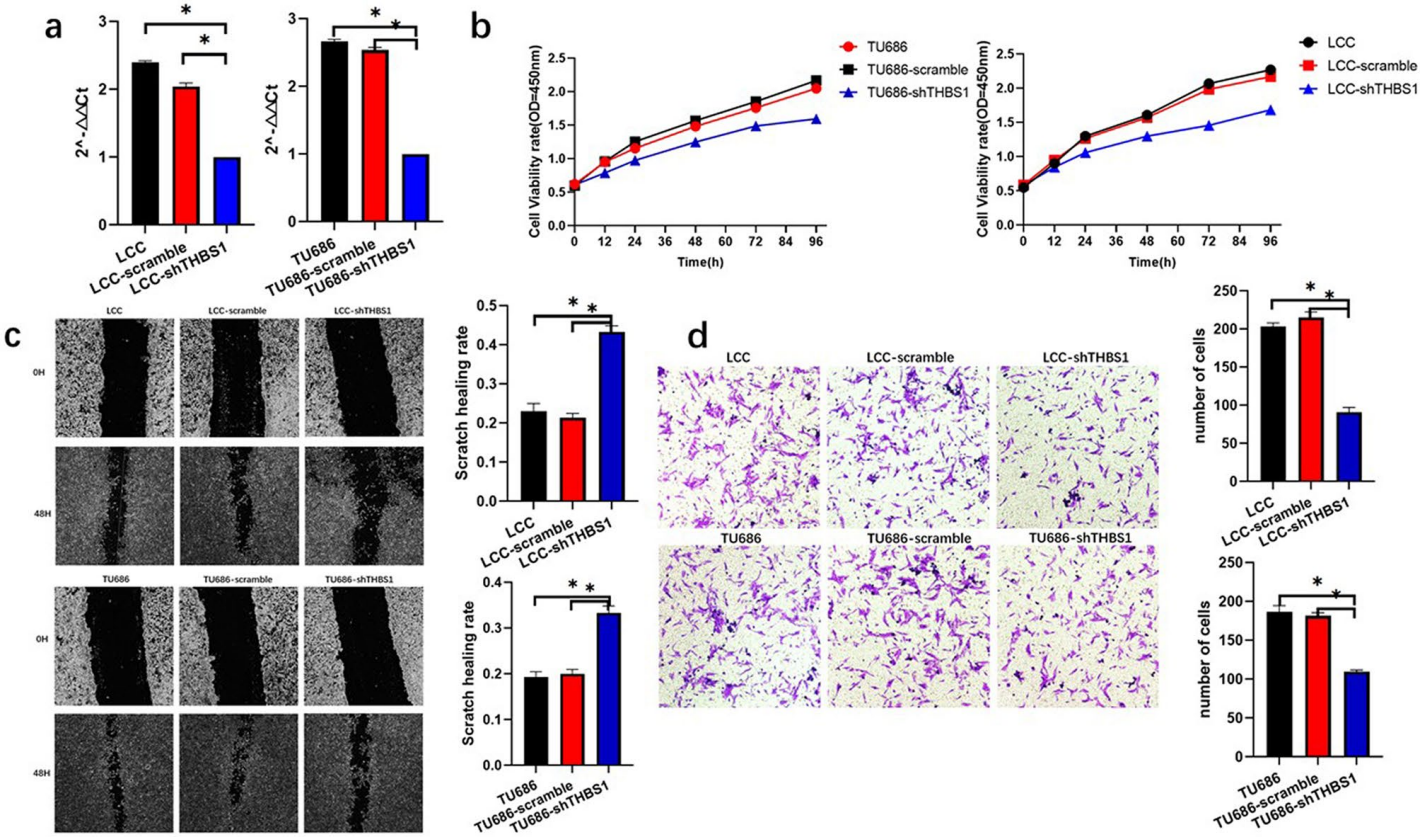
THBS1 promotes the proliferation, migration and invasion of laryngeal cancer tumor cells. (**a**) Construction of knockdown cell lines of THBS1 in laryngeal cancer cells LCC and TU686. (**b**) Knockdown of THBS1 can inhibit the proliferation level of laryngeal cancer tumor cells. (**c**) THBS1 knockdown can inhibit the migration level of laryngeal cancer tumor cells. d Knockdown of THBS1 can inhibit the invasion level of laryngeal cancer tumor cells.

The function of THBS1 was validated using in vitro cellular assays. We constructed THBS1 knockdown cell stable transfer lines with two cell lines, LCC and TU686, from a laryngeal carcinoma and validated them using PCR (Fig. 9a). We verified the proliferative ability of THBS1 using the CCK-8 assay. We found that the proliferative capacity of shTHBS1 was significantly weaker than that of the control cells, indicating that THBS1 was able to promote cell proliferation (Fig. 9b). We verified the migratory ability of THBS1 cells using a cell scratch assay and found that the migratory ability of shTHBS1 cells was weaker than the control cells, suggesting that THBS1 was able to promote cell migration (Fig. 9c). We verified the invasive ability of THBS1 cells using a transwell assay and found that the invasive ability of shTHBS1 was significantly weaker than that of the control cells, indicating that THBS1 can promote cell invasion (Fig. 9d). In summary, knockdown of THBS1 inhibited laryngeal cancer cell migration, invasion, and proliferation, suggesting that THBS1 can promote the development of laryngeal cancer cells.

## Discussion

Laryngeal cancer is one of the most common cancers of the respiratory system. Laryngeal squamous cell carcinomas account for approximately 98% of all laryngeal cancer cases. Despite advances in the integrative management of patients with laryngeal cancer, the 5-year OS has declined over the past 50 years because of insidious symptoms and the lack of effective early diagnostic methods^14^. Recently, a growing body of evidence suggested that the prognosis and chemotherapeutic outcomes of tumor patients are largely influenced by immune cell infiltration. Thus, the identification of new potential biomarkers and their association with immune infiltration has the potential to improve the prognosis of patients with laryngeal cancer and guide individualized treatment^1^. The lack of robust prognostic markers and low long-term survival rates for laryngeal cancer means that we need to find promising biomarkers to guide prognostic management and treatment choices. Owing to drastic advances in high-throughput technologies, such as RNA-seq whole genome sequencing, genetic analysis combined with powerful statistical algorithms has emerged as an effective strategy for screening molecular biomarkers for cancer diagnosis and treatment^15,16^.

Cells of cancer rely on alternative metabolic pathways to obtain or produce nutrients for survival^17^. Alongside the “Warburg effect,” fatty acids are also considered important players in tumor progression^18^. Fatty acids are essential energy sources and cellular components^19^. Evidence that fatty acid metabolism contributes profoundly to different stages of human carcinogenesis, including head and neck squamous cell carcinoma, is accumulating^20^. In our study, we determined key FAMRGs that were differentially expressed in laryngeal cancer to adequately predict prognosis using an integrated bioinformatics and experimental approach. Exploring the role of different fatty acid metabolic patterns in laryngeal cancer can help understand the role of fatty acid metabolism in the progression of laryngeal cancer. Metabolomic profiling can provide a greater understanding of the molecular pathways of laryngeal cancer and offers great potential for the discovery of new therapeutic approaches for laryngeal cancer.

We first screened FAMRG. Through the study of gene expression levels in the TCGA database, we screened 120 differentially expressed fatty acid metabolism genes, of which 51 genes were down-regulated and 69 genes were up-regulated. Combined with the prognostic information of laryngeal cancer patients in the GEO and TCGA databases, 9 genes related to the prognosis of laryngeal cancer, ACAA1, ACOT9, NCAPH2, NTHL1, CROT, ACSM3, SMS, EPHX2 and PON2, were screened out after merging the information. We constructed a sample risk score model based on these nine fatty acid metabolism genes with the following formula: risk score = ACAA1 × (–0.261903442393461) + ACOT9 × (0.269678824841202) + NCAPH2 × (–0.9101717202971) + NTHL1 × (–0.470406427009571) + CROT × (–0.411094477337431) + ACSM3 × (–0.517552679058424) + SMS × (0.577140908056887) + EPHX2 × (–0.21327197630715) + PON2 × (0.403413168237389).

This is the first study to explore the relationship between laryngeal cancer and genes related to fatty acid metabolism. To better understand the role of these genes in laryngeal cancer, we used the prognostic risk score model to predict the OS of patients with laryngeal cancer and divided the samples into high- and low-risk score groups based on the risk scores in the training set. Both the TCGA and GEO confirmed that samples in the high-risk score group had a worse prognosis than those in the low-risk score group. Furthermore, we plotted the time-related ROC curve at 1, 3, and 5 years to verify the accuracy of the prognostic risk score model. However, the association between laryngeal cancer and fatty acid metabolism remains unclear; meanwhile, our risk profile may provide clues in this area of research. Our results suggested that fatty acid metabolism may be involved in laryngeal cancer progression.

We further compared differences in response to drug therapy between different subgroups of patients to learn about the prognostic risk score model in laryngeal cancer. We found differences between the high-risk and low-risk groups for 22 drugs. In the GSE25727 cohort, there was a strong association between chemotherapy sensitivity and risk score based on RFS.

To further explore the potential role of fatty acid metabolism, we evaluated the correlation between immune cell populations and the risk profile in laryngeal cancer. We assessed the difference in immune cell infiltration between the high- and low-risk score groups in the TCGA. Immunosuppressive cell infiltration was abundant in the high-risk score group, with memory CD4-activated T-cell activation, CD8 + T cells, plasma cells, and T follicular helper cell enrichment. We evaluated the differences in immune checkpoint-related gene expression between the low-risk and high-risk score subgroups of the TCGA cohort in response to PDCD1, LAG3, CTLA4, and TIGHT treatment. We found that low-risk laryngeal cancer samples were more sensitive to PDCD1, LAG3, CTLA4, and TIGHT, indicating that fatty acid metabolism is of interest in terms of assessing the TME characteristics in patients with laryngeal cancer. These findings will provide new perspectives for exploring the metabolic mechanisms and treatment of laryngeal cancer.

In addition, we re-analyzed each sample in the TCGA database through the model composed of these 9 fatty acid metabolism genes, and scored each sample. Combining all gene expression profiles of laryngeal cancer samples in the TCGA database, we screened out 24 high-risk genes. Using Cytoscape software to process and display PPI network data to analyze these 24 high-risk genes, we took the top five hub genes: F3, THBS1, SERPINE1, TIMP3 and ITGA5.

We combined the TCGA and GEO databases to validate the prognostic analysis, and found that only THBS1 was the only gene with prognostic significance in both databases at the same time. are closely related. THBS1 is a gene that has been poorly studied before, which caught our attention. At the same time, we simply verified the invasion and migration functions of THBS1 on cell lines through cell phenotype experiments. We speculate that THBS1 may regulate the occurrence and development of laryngeal cancer through fatty acid metabolism, but we still need to conduct in-depth mechanism research in the future.

Our study suggests that genetic risk markers related to fatty acid metabolism are potential metabolic markers in laryngeal cancer immunotherapy. THBS1, one of our FAMRGs, is a typical stromal cell protein^21^ that is unexpressed in most non-tumor adult tissues; however, it is significantly upregulated in repair, tumor formation, and injury^22^. THBS1 expression strongly correlates with poor prognosis. In this study, we explored specific differences in TME immune cell infiltration between patients with low and high THBS1 expression using the median THBS1 expression as a cutoff value. Tumors with high THBS1 expression showed increased infiltration of memory CD4 rest compared with those with low expression, whereas tumors with low THBS1 expression showed significantly increased infiltration of CD8 + T cells, plasma cells, dendritic cells, T follicular helper cells, and memory CD4-activated T cells, compared with those with high expression. Furthermore, we investigated the cellular function of THBS1 by constructing a THBS1 knockdown stable cell line for laryngeal carcinoma. We found that cell invasion, migration, and proliferation were significantly diminished after the knockdown of THBS1 in laryngeal cancer cells. This finding suggests that THBS1 promotes laryngeal cancer cell proliferation, invasion, and migration, which is consistent with our previous analysis of the TCGA database.

## Supplementary Information

Supplementary Information 1.

Supplementary Information 2.

Supplementary Information 3.

Supplementary Information 4.

## Data Availability

Transcriptomic and clinical data for all thyroid cancer samples were obtained from the TCGA database (https://portal.gdc.cancer.gov/). Expression information of all ARGs in thyroid cancer data was obtained from the Human Autophagy Database (HAD) (http://www.autophagy.lu/).

## Abbreviations

TCGA: The Cancer Genome Atlas
GEO: Gene Expression Omnibus
FAMRGs: Fatty acid metabolism-related genes
qRT-PCR: Quantitative real-time PCR
PPI: Protein-protein interaction
PCA: Principal component analysis
K-M: Kaplan-Meier
ROC: Receiver Operation Characteristic
GSVA: Gene Set Variation Analysis
CCK-8: Cell Counting Kit-8
GO: Gene ontology
KEGG: Kyoto Encyclopedia of Genes and Genomes
