# Author Correction: THBS1, a fatty acid-related metabolic gene, can promote the development of laryngeal cancer

**DOI:** 10.1038/s41598-022-25904-w

**Published:** 2022-12-13

**Authors:** Fei-Hong Ji, Xin-Guang Qiu

**Affiliations:** Thyroid Surgery, The First Afliated Hospital of Zhengzhou University, Zhengzhou, Henan China

Correction to: *Scientific Reports* https://doi.org/10.1038/s41598-022-23500-6, published online 05 November 2022

In the original version of this Article, author Fei-Hong Ji was incorrectly listed as a corresponding author. The correct corresponding author for this Article is Xin-Guang Qiu. Correspondence and request for materials should be addressed to fccqiuxg@zzu.edu.cn.

The original Article has been corrected.

